# Evidence of Long-range nerve pathways connecting and coordinating activity in secondary lymph organs

**DOI:** 10.1186/s42234-020-00056-2

**Published:** 2020-10-23

**Authors:** Victoria Cotero, Tzu-Jen Kao, John Graf, Jeffrey Ashe, Christine Morton, Sangeeta S. Chavan, Stavros Zanos, Kevin J. Tracey, Christopher M. Puleo

**Affiliations:** 1 General Electric Research, Niskayuna, NY USA; 2 Feinstein Institutes for Medical Research, Manhasset, NY USA

**Keywords:** Neuromodulation, Bioelectronic medicine, Immunology, Neuroscience, Neural immune reflexes, Biomedical engineering

## Abstract

**Background:**

Peripheral nerve reflexes enable organ systems to maintain long-term physiological homeostasis while responding to rapidly changing environmental conditions. Electrical nerve stimulation is commonly used to activate these reflexes and modulate organ function, giving rise to an emerging class of therapeutics called bioelectronic medicines. Dogma maintains that immune cell migration to and from organs is mediated by inflammatory signals (i.e. cytokines or pathogen associated signaling molecules). However, nerve reflexes that regulate immune function have only recently been elucidated, and stimulation of these reflexes for therapeutic effect has not been fully investigated.

**Methods:**

We utilized both electrical and ultrasound-based nerve stimulation to activate nerve pathways projecting to specific lymph nodes. Tissue and cell analysis of the stimulated lymph node, distal lymph nodes and immune organs is then utilized to measure the stimulation-induced changes in neurotransmitter/neuropeptide concentrations and immune cellularity in each of these sites.

**Results and conclusions:**

In this report, we demonstrate that activation of nerves and stimulated release of neurotransmitters within a local lymph node results in transient retention of immune cells (e.g. lymphocytes and neutrophils) at that location. Furthermore, such stimulation results in transient changes in neurotransmitter concentrations at distal organs of the immune system, spleen and liver, and mobilization of immune cells into the circulation. This report will enable future studies in which stimulation of these long-range nerve connections between lymphatic and immune organs can be applied for clinical purpose, including therapeutic modulation of cellularity during vaccination, active allergic response, or active auto-immune disease.

## Introduction

In recent years, several nerve reflexes have been described that modulate the function of the immune system. These include the vagus nerve-mediated anti-inflammatory reflex, that alters cytokine release from macrophages (Chavan & T., 2017; Wang et al., 2002; Gunasekaran et al., 2018; Tracey, 2009; Tracey, 2016; Borovikova et al., 2000) and modulates circulating neutrophil activity (Huston et al., 2009), adrenal reflexes that modulate systemic inflammation via epinephrine, glucocorticoids, or dopamine (Torres-Rosas, 2014; Cain & Cidlowski, 2017; Mracsko et al., 2014), a central nervous system (CNS) associated reflex that modulates migration of leukocytes across the blood-brain barrier (Tanaka et al., 2017), and an intestinal reflex that regulates the activity of macrophages within intestinal epithelium (Matteoli et al., 2014). However, despite the common use of nerve stimulation devices as a non-pharmaceutical therapeutic option for cardiovascular, musculoskeletal, gastrointestinal, and urinary system pathologies, there are few examples of the use of these medical devices in immunological applications (Koopman et al., 2016).

It is widely accepted that alterations of immune cells, in terms of absolute numbers and of their activation state, within tissues are driven primarily by inflammation-mediated mechanisms. Cytokines and other host- or pathogen-derived inflammatory molecules drive changes in the expression of cellular adhesion molecules and chemokines, which alter both the migratory activity of immune cells and the permeability of tissue barriers. In turn, the distribution and activation state of immune cells within a specific tissue alters the immune response, including response to vaccination, immunotherapy, infection, or allergens (Luster et al., 2005). Τhere is increasing evidence that the nervous system plays an important role in both homeostatic maintenance and stimulus-elicited changes in immune cell distribution (Gunasekaran et al., 2018; Tracey, 2014; Nakai et al., 2014). Immune cells exhibit cell-type specific differential expression of neurotransmitter receptors, including alpha or beta adrenergic receptors, nicotinic acetylcholine receptors (Scanzano & Cosentino, 2015). Further, immune cell egress from lymph nodes is modulated by a functional association between adrenergic and chemokine receptors (Nakai et al., 2014), as stimulation of lymphocyte adrenergic receptors has been shown to promote chemotaxis in response to chemokines CCR7 or CXC4, and pharmacological blockade of the chemokine receptors inhibited this effect. Several reports showed that lymphocyte concentrations within the lymph node and blood compartments follow a diurnal rhythm, and depletion of adrenergic nerves inhibits diurnal variation in adaptive immune response (Druzd et al., 2017). These pharmacological studies have been further validated through detailed nerve tracing and immunohistochemical studies of lymph organs, which demonstrate innervation in two major locations, the T cell zones and the entrance/exit areas (e.g. subsinoidal layer of lymph nodes and splenic white pulp and subepithelial dome of Peyer’s patches) (Scanzano & Cosentino, 2015). Potential clinical utility of such neuro-immune interactions has been shown in initial studies that demonstrated elevated antigen-specific antibody titers after immunizations in the morning, during periods of high sympathetic tone, versus in the afternoon, periods of low sympathetic tone (Druzd et al., 2017; Long et al., 2016) This result is further supported by enhancement of germinal center B cells and follicular helper T cells within the draining lymph node in cohorts immunized during circadian periods of high sympathetic activity, and demonstration of increased lymphatic antigen flow in the draining lymph nodes of subjects treated locally with a nerve blocking agent (Long et al., 2016).

Despite the solid pharmacological, histochemical, and physiological evidence of nerve reflex influence over whole body lymphocyte distribution, only few studies have investigated targeted activation of these neural reflexes to promote a specific immune cell response (Tracey, 2014). There are no investigations into the potential for nerve mediated signaling between the lymph node compartment and other major immune cell stores within distal immune organs like the spleen, liver, and thymus. Herein, we demonstrate that electrical and ultrasound-based (Cotero et al., 2019a; Cotero et al., 2019b; Puleo et al., 2019) sciatic nerve activation at the site of its entry in a specific lymph node (LN) results in accumulation of both lymphocytes and neutrophils within that local LN compartment, i.e. at the site of stimulated nerve activity, but not in distal LNs. The local effect of nerve stimulation on immune cell trafficking is dependent on the voltage and frequency of the stimulus and correlates with a stimulus-induced increase in local neurotransmitter concentrations. In addition, nerve stimulation resulted in additional nerve-mediated changes in cellularity (i.e. tissue concentration of immune cells) in the spleen and liver, which correlated with release of different neurotransmitters in each compartment (i.e. epinephrine in the spleen, but both epinephrine and norepinephrine in the liver). The effect of stimulation on immune cell trafficking in these distal immune organs was attenuated by severing the sciatic nerve above the stimulating electrodes, thereby disrupting afferent signaling from the stimulus site. We observed different kinetics in the effect on immune cell trafficking for neutrophils vs. lymphocytes, and for spleen vs. liver. Finally, we produced the effect on immune cell trafficking using a non-invasive, ultrasound-based (Cotero et al., 2019a; Cotero et al., 2019b; Puleo et al., 2019) nerve stimulating device. These results provide evidence that activation of neural reflexes by nerve stimulation can modulate whole body immune cell distribution; they also demonstrate the use of invasive and non-invasive neuromodulation tools and devices to activate these reflexes in future clinical applications.

## Methods

### Electrical stimulation system and methods

Electrical stimulation was performed with either a voltage source stimulator or a current source stimulator. Studies were performed using stainless steel needles applied directly to the surgically exposed sciatic nerve in bipolar configuration.

#### Electrodes

Electrodes were constructed using a fixture to hold two 22 mm long by 0.18 mm diameter stainless steel needles (Millennia Sterile Accupuncture needle; 0.18mmx25mm) approximately 5 mm apart. The needles were insulated by covering the surfaces with epoxy (Kwik-cast) leaving approximately 5 mm of electrode tips exposed as a conducting surface. One electrode was connected to the positive terminal of the stimulator and the other electrode connected to the negative terminal of the stimulator. (see Supplemental Fig. 4).

#### Stimulator circuit

The voltage source stimulator consisted of a function generator (Agilent 33120A) with an internal source resistance of 50 Ω, programmed to output a waveform of 10 V, 7 V, 5 V, 2 V or 0.5 V with a pulse length of 50 msec at a repetition rate of 30 kHz, 200 Hz, 20 Hz or 0.5 Hz. The waveform was adjusted with a voltage offset to balance the net voltage-time between the positive (pulse) cycle and the negative (off) cycle. The current source stimulator consisted of a custom voltage to current circuit (see Supplemental Fig. S5) driven by an analog output of a data acquisition and analysis system (MP150, Biopac Systems, Goleta CA). The custom circuit provided a current output of approximately 1 mA per 1 V input. The custom circuit also provided output current monitoring and output voltage monitoring to the Biopac for analysis. A biphasic pulse was constructed with a positive output for 0.2 msec, no output for 0.2 msec, then a negative output for 0.2 msec (equal in magnitude to the positive output), followed by a period of no output which was adjustable to change the effective pulse repetition rate (for example, 49.4 msec for a 20 Hz repetition rate). (see Supplemental Fig. S6).

#### Electrode impedance analysis

The impedance of the electrodes as placed in tissue was evaluated for each of the positive current pulses. Over the course of 3 min, there are 3600 pulses delivered at a rate of 20 Hz. Using the monitored output current and output voltage as measured by the Biopac system, the electrode impedance was measured for 5 different rats in the electro-acupuncture configuration. The average electrode impedance ranged from 1200 to 1600 Ω. (see Supplemental Fig. S7).

### Ultrasound stimulation system and methods

A block diagram of the focused ultrasound (FUS) system has been shown previously (Cotero et al., 2020). The system consists of a 1.1 MHz, high intensity focused ultrasound (HIFU) transducer (Sonic Concepts H106), a matching network (Sonic Concepts), an RF power amplifier (ENI 350 L) and a function generator (Agilent 33120A). The 70-mm-diameter HIFU transducer has a spherical face with a 65-mm radius of curvature. It has a 20-mm-diameter hole in the center into which an imaging transducer can be inserted. The transducer depth of focus is 65 mm. The numerically simulated pressure profile has a full width at half amplitude of 1.8 mm laterally and 12 mm in the depth direction. The HIFU transducer is acoustically coupled to the animal through a 6-cm-tall plastic cone filled with degassed water. A function generator produces a pulsed sinusoidal waveform, and this pulsed sinusoidal waveform is amplified by the RF power amplifier and sent to the impedance-matching network connected to the HIFU transducer. The pulse center frequency was 1.1 MHz, the pulse repetition period was 0.5 ms (corresponding to a pulse repetition frequency of 2000 Hz). The voltage-to-pressure calibration of the HIFU transducer was performed in degassed water using a needle hydrophone (ONDA HNA-0400). The HIFU transducer was driven by a 100-cycle sinusoidal voltage waveform. To locate the position of peak pressure, the hydrophone was scanned in a neighborhood of the nominal transducer focus point in 0.1 mm steps in the lateral plane and in 0.2 steps in the depth direction. For driving voltages below 60 V, the nonlinearity of water was small, i.e., the maximum negative pressure and the maximum positive pressure were nearly equal, and the pressure varied linearly with driving voltage; the applied drive voltage required for nerve stimulation has been reported previously (Cotero et al., 2020). A Vivid E9 ultrasound system (GE Healthcare) or an 11 L probe (GE Healthcare) were used for the ultrasound scan before neuromodulation started. The imaging beam of the probe was aligned with the U/S stimulating beam. Therefore, one could confirm that the U/S beam was targeted at the region of interest using an image of the targeted organ/lymph node (visualized on the Vivid E9).

### Animal models, tissue excision, and molecular methods

Adult male Sprague–Dawley rats 8–12 weeks old (250–300 g; Charles River Laboratories) were housed at 25 °C on a 12-h light/dark cycle and acclimatized for 1 week, with handling, before experiments were conducted to minimize potential confounding measures due to stress response. Water and regular rodent chow were available ad libitum. Experiments were performed under protocols approved by the Institutional Animal Care and Use Committee of GE Global Research.

#### Electrical stimulation protocol

Prior to stimulation the rat was anesthetized with 1–2% isoflurane and laid prone on a water circulating heating pad. Methylene blue (0.5 mg/kg of a 1% dye solution) was injected into the foot pad of the rat to trace the lymphatic and highlight the popliteal lymph node prior to surgical exposure and nerve stimulation. To gain access to both the sciatic nerve and visualize the popliteal fossa, a S-shaped incision was made through the bicep femoris exposing the popliteal fossa and sciatic nerve. The surgical area is irrigated in sterile saline to prevent damage from excessive drying of the tissue during the experiment. A stainless steel electrode, described above, were placed along the sciatic nerve nearest to the sacral plexus region of the spine. Following electrode placement, a pulse is applied for 3 min. Following stimulation, the area is irrigated once more with sterile saline and the surgical flap replaced and sutured closed. The animal is then maintained under anesthesia for the duration of the study designated incubation period.

#### Ultrasound stimulation protocol

The region above the designated point for U/S stimulus was shaved with a disposable razor and animal clippers prior to stimulation. A Vivid E9 Diagnostic imaging ultrasound system was used to identify the region of interest as described above. The area was marked with a permanent marker for later identification. Ultrasound stimulation was applied using a the HIFU system. The U/S probe was placed at the designated area of interest identified by the diagnostic ultrasound probe. An U/S stimulus was then applied with total duration of a single stimulus not surpassing a single 1 min pulse. At no point was the energy allowed to reach levels associated with thermal damage and ablation/cavitation (35 W/cm^2^ for ablation/cavitation).

#### LPS exposure

Rodents were anesthetized with 2–4% isoflurane prior to IP administration of an LD75 dose of LPS (10 mg/kg). Following injection, animal were then allowed to incubate under lower anesthesia (1.5–2% isoflurane). Throughout the study, the level of anesthesia is monitored through assessment of either deep pain recognition (pedal reflex, pinna reflex) to confirm deep anesthesia or corneal response during the incubation period. During incubation period, low level anesthesia was maintained to reduce discomfort associated with LPS induced inflammation, which may trigger changes in stress response altering blood chemistries; however, isoflurane was maintained at superficial levels to prevent reduced cardiac output and hypothermia induced by isoflurane.

After incubation (1 h), the animal was euthanized and tissue, blood samples are collected as described below. Endotoxin (lipopolysaccharide (LPS) from *Escherichia coli*, 0111: B4; Sigma–Aldrich) was used to produce a significant state of inflammation in naive adult Sprague–Dawley rats prior to neuroimmune stimulation. LPS was administered to animals (10 mg/kg), which corresponds to an approximate LD75 dose.

#### Nerve blocking

Animals were anesthetized with 2–4% isoflurane and depth of anesthesia confirmed through assessment of deep pain recognition as described above. For lidocaine injection into the sciatic nerve, the rat was manually restrained in a lateral recumbent position with the hindlimb to be injected held at a right angle with the longitudinal axis of the trunk. A single fine tipped insulin syringe was then used to administer a 1% dose of lidocaine (100uL; Sigma Aldrich, pH 6.4) or isotonic saline to the sciatic nerve located caudal to the greater trochanter. This method of sciatic nerve block has been assessed in the literature (Thalhammer et al., 1995). Furthermore, use of the fine gauge syringe left no indication of trauma.

### Tissue harvesting and sample preparation

At the completion of the study animals were deeply anesthetized with isoflurane (5% isoflurane) and reaction tested by corneal reflex and toe pinch. Following confirmation of complete anesthesia, the animal is laid in a dorsal recumbent position and a V-cut made through both the skin and abdominal wall, caudal to the last rib. Internal organs are then moved, and a needle inserted through the diaphragm into the vena cava and blood drawn out for a total volume of 5-9 mL to confirm exsanguination. Freshly collected blood was used in cell-counting studies immediately following collection. The remainder of the collected blood was stored in EDTA collection tubes and stores in -20C. Following exsanguination, organs (including lymph nodes, spleen, liver, thymus) were rapidly removed and homogenized in a solution of phosphate-buffered saline (PBS), containing phosphatase (0.2-mM phenylmethylsulfonyl fluoride, 5-μg/mL aprotinin, 1-mM benzamidine, 1-mM sodium orthovanadate, and 2-μM cantharidin) and protease (1-μL to 20 mg of tissue as per Roche Diagnostics) inhibitors. A targeted final concentration of 0.2-g tissue per mL PBS solution was applied in all samples. Lymphatic fluid samples were filtered through a 70um cell strainer (Corning) prior to cell counting. Samples were stored at − 80 °C following cell counting.

### HPLC analyses

Tissue homogenates were initially homogenized with 0.1-M perchloric acid and centrifuged for 15 min, after which the supernatant was separated, and the sample injected into the HPLC. Catecholamines norepinephrine and epinephrine were analyzed by HPLC with inline ultraviolet detector. The test column used in this analysis was a Supelco Discovery C18 (15-cm × 4.6-mm inside diameter, 5-μm particle size). A biphasic mobile phase comprised of [A] acetonitrile: [B] 50 = mM KH_2_PO_4_, set to pH 3 (with phosphoric acid). The solution was then buffered with 100-mg/L EDTA and 200-mg/L 1-octane-sulfonic acid. Final concentration of mobile phase mixture was set to 5:95, A:B. A flow rate of 1 mL/min was used to improve overall peak resolution while the column was held to a consistent 20 °C to minimize pressure compaction of the column resulting from the viscosity of the utilized mobile phase. The UV detector was maintained at a 254-nm wavelength, which is known to capture the absorption for catecholamines including norepinephrine, epinephrine, and dopamine.

For ultrasound stimulation, animals were anesthetized with 2–4% isoflurane and laid on a water circulating warming pad to prevent hyperthermia during the procedure. Prior to neuromodulation, the area above the anatomical area of interest was shaved with a disposable razor and animal hair clippers. After targeting (as described above), the ultrasound stimulus was applied for a duration of 1 min. The LPS was then administered immediately following the first ultrasound stimulus (as described above). A second 1-min ultrasound stimulus was then applied following the LPS administration for a total duration of 2 min. The animal was then allowed to incubate under anesthesia, and blood samples were taken as described above. After incubation, the animal was euthanized, and tissue and blood samples were taken as described above.

### Lymphatic fluid collection

Prior to euthanization, rodents were deeply anesthetized, and a single incision was made through the abdominal wall and the superior mesenteric lymph duct located through identification of anatomical landmarks (~ 0.5-1 mm in diameter, located perpendicular to the right kidney and parallel to the mesenteric artery) and completely exposed. Following isolation of the lymph duct, a small hole was made with fine tip IRIS scissors and a cannula inserted into the lymph duct.

### Cell counting assays

Cell counting was performed on a Hemavet 950 analyzer (Drew Scientific). A 200uL aliquot of lymphatic fluid or tissue homogenate was mixed on a rotary mixer immediately following collection for a minimum of 10 min at RT. Analysis of the sample occurred within no more than 1 h post collection. No cell count analysis was performed on previously frozen samples. Hematological assessment of each sample including white blood cell, neutrophil and lymphocytes were performed in accordance with instrument supplier documentation and normalized to the weight of the collected tissue sample.

### Statistical analysis

Animal group sizes for each experiment were estimated using a desired power of 0.9 based on prior studies accounting for a minimum group size of *n* = 5 for each study. However, experimental variability, suspected to originate from animal-to-animal variation resulted in the increase in group size in several studies, as indicated in the corresponding figure legends. All data were expressed as means ± SE. Statistical analysis was performed using a Student’s t-test and Mann-Whitney post hoc or one-way analysis of variance (ANOVA) with a Tukey’s post hoc analysis. Statistical significance is indicated as a * (for *P* < 0.05), ** (for *P* < 0.005) and *** (for *P* < 0.0005).

## Results

### Effect of direct sciatic nerve stimulation on neurotransmitter release

**Fig. 1:**
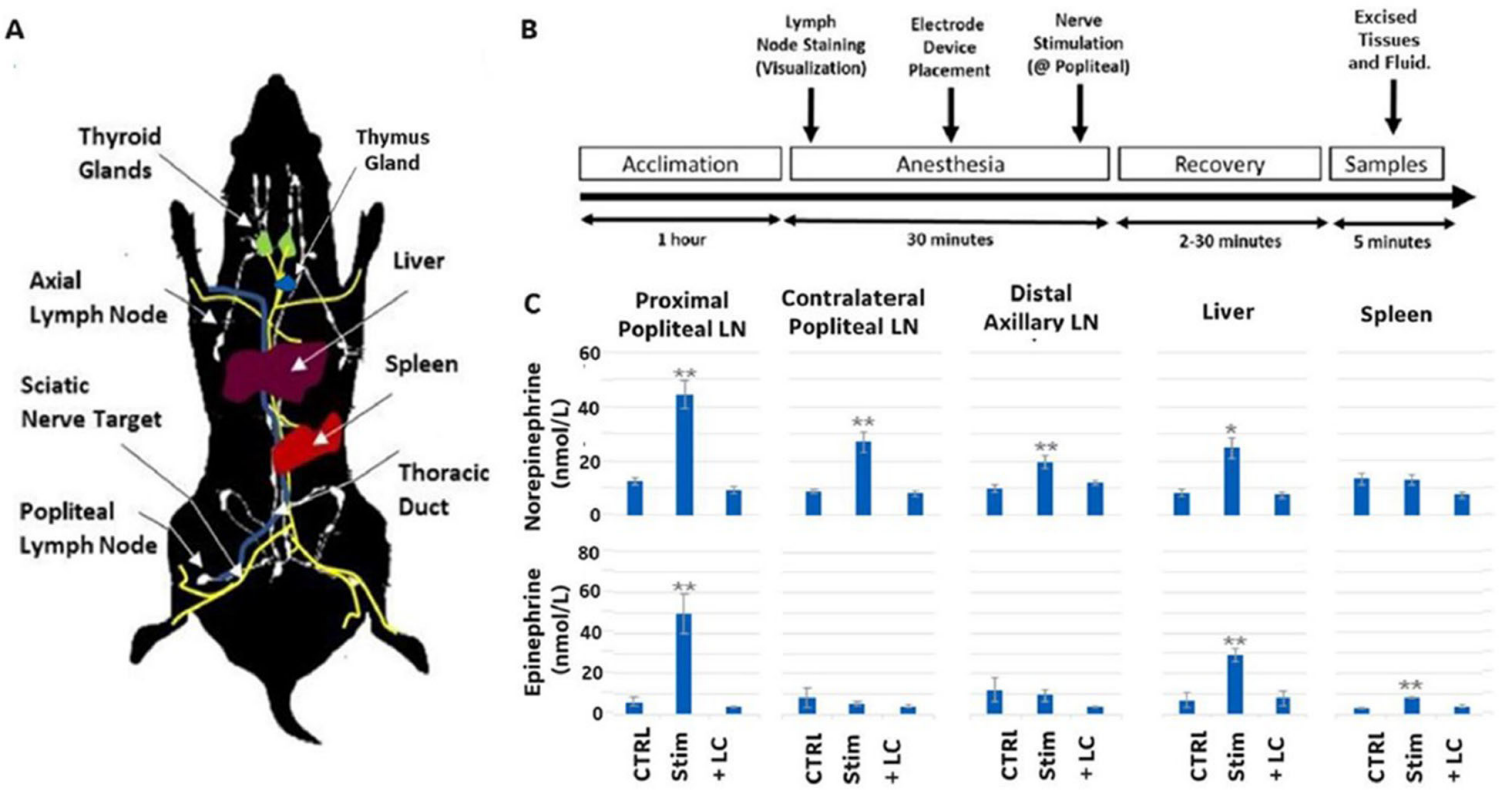
**a** A schematic diagram of the primary and secondary lymph organs in the rat, along with potential paths of the lymphatic ducts and associated connecting nerves (green- thyroid glands, purple- liver, red- spleen, blue- lymphatic duct, white- lymph nodes and lymphatic ducts, yellow- lymph associated nerves). **b** A timeline of the experimental procedure for lymph node nerve stimulation and subsequent tissue excision and analysis. **c** Normalized concentration of norepinephrine and epinephrine in excised tissues with (Stim) and without (sham control; CTRL) electrical sciatic nerve stimulation (see materials and methods for stimulation details), or with electrical stimulation following local lidocaine administration (+LC). *N* = 7. One way ANOVA with multiple comparison analysis * = *p* ≤ 0.05, ** = *p* ≤ 0.005,*** = *p* ≤ 0.0005; Electrical stimulation parameters: 0.5 mA, 20 Hz, pulse length 50 msec

Figure 1a shows a schematic of the lymphatic system within the rat and associated primary and secondary immune organs (i.e. spleen, lymph nodes, liver, and thymus). The popliteal lymph node (LN) was chosen as the local site of stimulation, as injection of dye within lymphatic structures in the foot pad enabled visualization of the entire lymphatic chain within the leg. This enabled visualization of popliteal LN and targeting of the sciatic nerve bundle entering the lymphatic chain. Figure 1b shows a timeline of the immune cell trafficking experiments, including stimulation and tissue sampling times. A bipolar electrode (Toda & Ichioka, 1979) was placed across the nerve bundle through a small incision and the animals received a three minute stimulation with electric pulses of 0.5–3000 Hz with a current between 0 and 10 mA (see materials and methods for additional information on electrical pulse settings and analysis). Figure 1c shows the resulting neurotransmitter concentrations in various lymph compartments after stimulation (using a stimulation parameter of 0.5 mA at 20 Hz). Direct electrical stimulation of the sciatic nerve resulted in the significant increase of epinephrine and norepinephrine in the proximal popliteal LN (i.e. the lymph node closest to the site of stimulation) compared to the non-stimulated control (Fig. 1c) or when nerve conduction was blocked by administration of lidocaine (LC) at the electrode site prior to stimulation. The neurotransmitters and neuropeptides measured were those previously shown to alter immune cell trafficking or activity (Tracey, 2016; Torres-Rosas, 2014; Tracey, 2014; Scanzano & Cosentino, 2015; Druzd et al., 2017; Lorton & Bellinger, 2015; Gonzalez et al., 2011; Pongratz & Straub, 2014). Interestingly, neurotransmitter concentrations were also modulated in other distal immune sites. Within the contralateral popliteal lymph node (i.e. in the other non-stimulated leg) norepinephrine was increased post-stimulation, but epinephrine was not. However, in the distal axillary lymph node no increase in either neurotransmitter was observed. In the liver there was a stimulation-induced increase in both neurotransmitters, but in the spleen the increase was only observed in epinephrine. Supplemental Fig. 1 and 2 shows additional measures of neurotransmitter and neuropeptide concentrations taken from the excised proximal popliteal lymph node 5 min after stimulation, for various stimulation frequencies and intensities. The data showed an epinephrine response across a wider range of stimulation frequencies (0.5–200 Hz) as compared to norepinephrine, which showed a response at only 20 Hz pulsing frequency. Using the 20 Hz stimulation frequency, there was a range of responses across the stimulation intensities. Epinephrine, norepinephrine, and neuropeptide Y (NPY) concentrations within the proximal LN increased as intensity was decreased from 10 to 0.5 mA (with a maximum percent change compared to control at 0.5 mA. Dopamine concentrations did not significantly change at low stimulus intensity, and concentration of substance P and vasoactive intestinal peptide (VIP) did not change at any of the applied intensities. Based on these results, stimulation parameters of 0.5 mA at 20 Hz were used for subsequent experiments.

### Effects of direct sciatic nerve stimulation on WBC counts

**Fig. 2:**
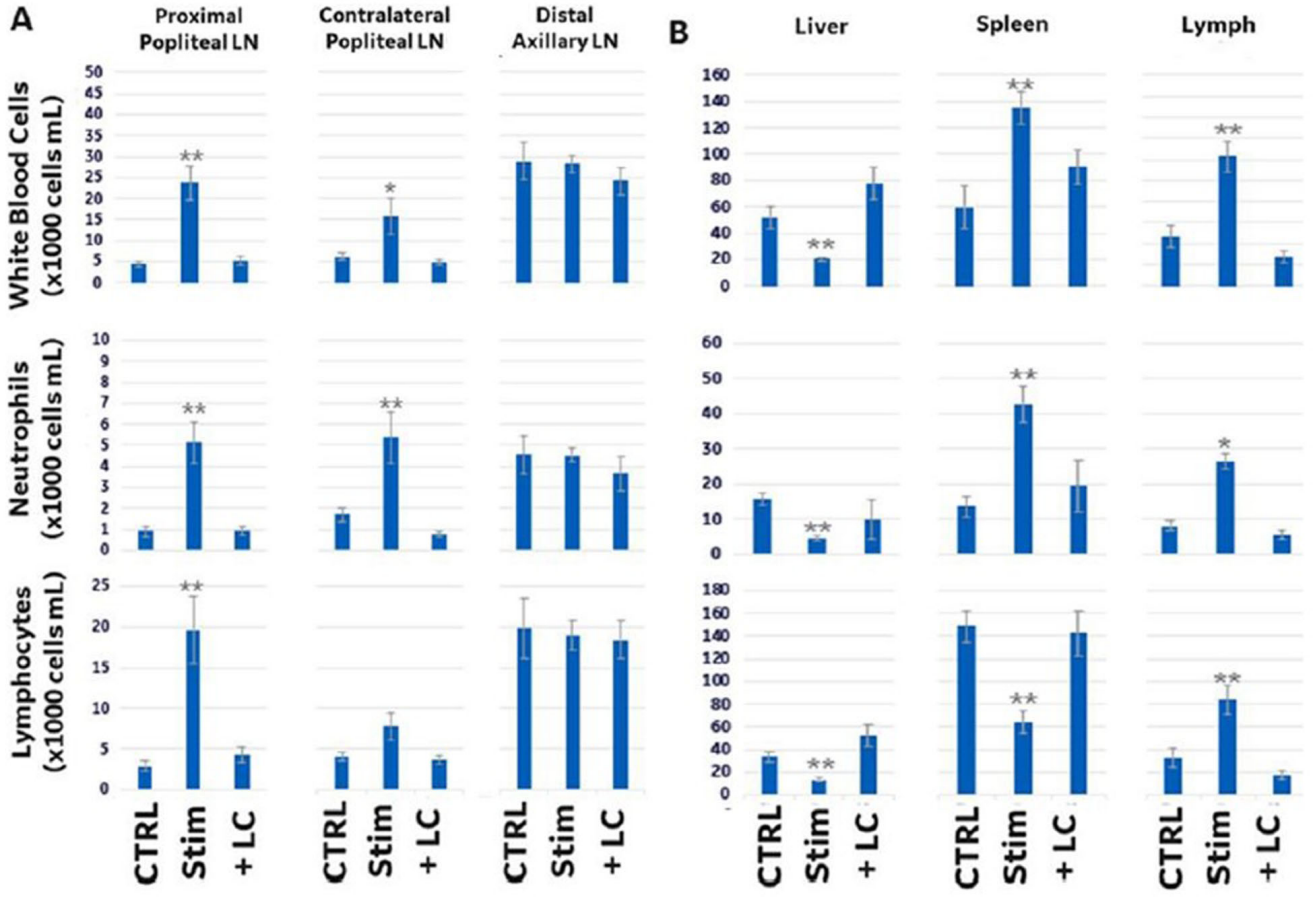
**a** Total white blood cell (WBC), lymphocyte (LY), and neutrophil (NE) numbers within the excised lymph nodes (see materials and methods for tissue collection and analysis details). The popliteal lymph node directly adjacent to the sciatic nerve stimulation site was collected and analyzed (proximal popliteal LN), along with the contralateral popliteal lymph node from the non-stimulated leg (contralateral popliteal LN) and a distal axillary lymph node (distal axillary LN). **b** Total white blood cell (WBC), lymphocyte (LY), and neutrophil (NE) numbers within immune sites distal to the stimulation site, including the liver, spleen, and lymphatic fluid (collected from the lymphatic duct). *N* = 9. One way ANOVA with multiple comparison analysis * = *p* ≤ 0.05, ** = *p* ≤ 0.005,*** = *p* ≤ 0.0005; Electrical stimulation parameters: 0.5 mA, 20 Hz, pulse length 50 msec

**Fig. 3:**
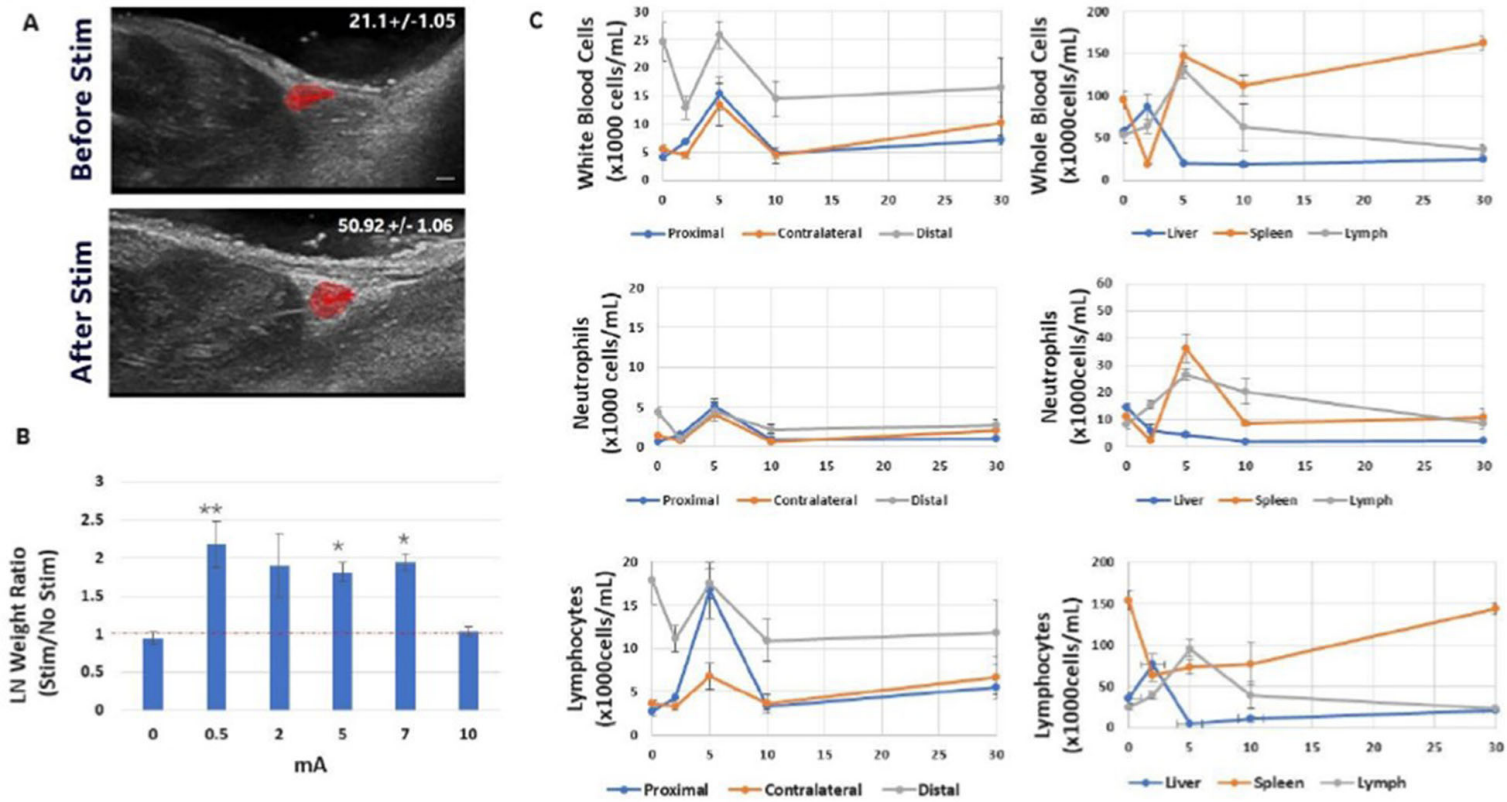
**a** An ultrasound image of the same popliteal lymph node (LN) before and after electrical stimulation of the sciatic nerve directly above the LN site. The lymph node has been highlighted and the diameter of the lymph node before and after stimulation is shown. **b** The total weight of excised lymph nodes without (0 mA; sham control) or with electrical stimulation at different stimulation intensities (0.5, 2, 5, 7.5, 10 mA; using stimulation frequency of 20 Hz with 50 msec pulse length). **c** (left panels) The concentration of white blood cells (WBC), neutrophils (NE), or lymphocytes (LY) taken from the stimulated (direct LN) and other distal lymph nodes (contralateral popliteal and axillary LN) at different times after electrical stimulation of the sciatic nerve above a popliteal lymph node (0, 2, 5, 10, and 30 min following stimulation). (right panels) The concentration of white blood cells (WMC), neutrophils (NE), and lymphocytes (LY) taken from the distal liver, spleen, and lymphatic fluid/lymphatic duct sites at different times after electrical stimulation of the sciatic nerve above a popliteal lymph node (0, 2, 5, 10, and 30 min following stimulation). N = 9. One way ANOVA with multiple comparison analysis * = *p* ≤ 0.05, ** = *p* ≤ 0.005,*** = *p* ≤ 0.0005; Electrical stimulation parameters: 0.5 mA, 20 Hz, pulse length 50 msec

Figure 2 A and B show that, in addition to neurotransmitter changes, nerve stimulation also produced changes in the number of white blood cells within the sampled immune tissue sites. Like neurotransmitter response, the immune cell types driving cellularity changes was different in each immune organ. A significant increase in both lymphocytes and neutrophils was observed in the targeted proximal popliteal LN, whereas only neutrophils were affected in the contralateral popliteal LN and no change in cellularity was observed in the distal lymph node. In the liver, there was an overall decrease in white blood cell counts, which involved both lymphocytes and neutrophil populations. In the spleen there was an overall increase in white blood cell concentrations due to an increase in neutrophils, despite a decrease in splenic lymphocyte numbers. Analysis of cell populations in fluid from the lymphatic duct showed that mobilization of cells from both the liver and spleen (and/or other immune cell store sites not measured such as blood, skin, or intestines) resulted in a significant increase in circulating cells of both types within the lymph/lymphatic fluid. As shown by others (Nakai et al., 2014), modulation of the cellularity within the proximal popliteal LN resulted in a measurable difference in size of the lymph node compared to non-stimulated controls. Figure 3a shows ultrasound images of a popliteal LN before and after stimulation; an increase in the size of the LN is evident. This overall size change was also validated by measuring the weight of the excised LN, which showed an increase compared to non-stimulated controls (Fig. 3b). Both images and lymph node tissue samples were harvested within 5 min of stimulation for size and weight measurements. Figure 3c shows that these changes in immune cell populations were transient in the LNs, since cellularity in both LNs returned to pre-stimulus values within 10 min. Lymphocyte and neutrophil counts returned to pre-stimulus levels within 20 min in the liver, and they remained elevated for at least 30 min in the spleen (Fig. 3c)

Local lidocaine application at the nerve prior to stimulation resulted in attenuation of all stimulation-associated neurotransmitter changes (Fig. 1c), and corresponding changes to immune cell counts in the LN, liver and spleen (Fig. 2b and c). In contrast, resection of the sciatic nerve rostral to the stimulation electrode (supplemental Fig. 3) resulted in attenuation of changes in immune cell counts only in the spleen and contralateral LN, with no significant effect in the proximal popliteal LN. In addition, nerve resection attenuated stimulation-associated decrease in lymphocytes but not neutrophils in the liver.

### Effects of LN-focused ultrasound neurostimulation on WBC counts

**Fig. 4:**
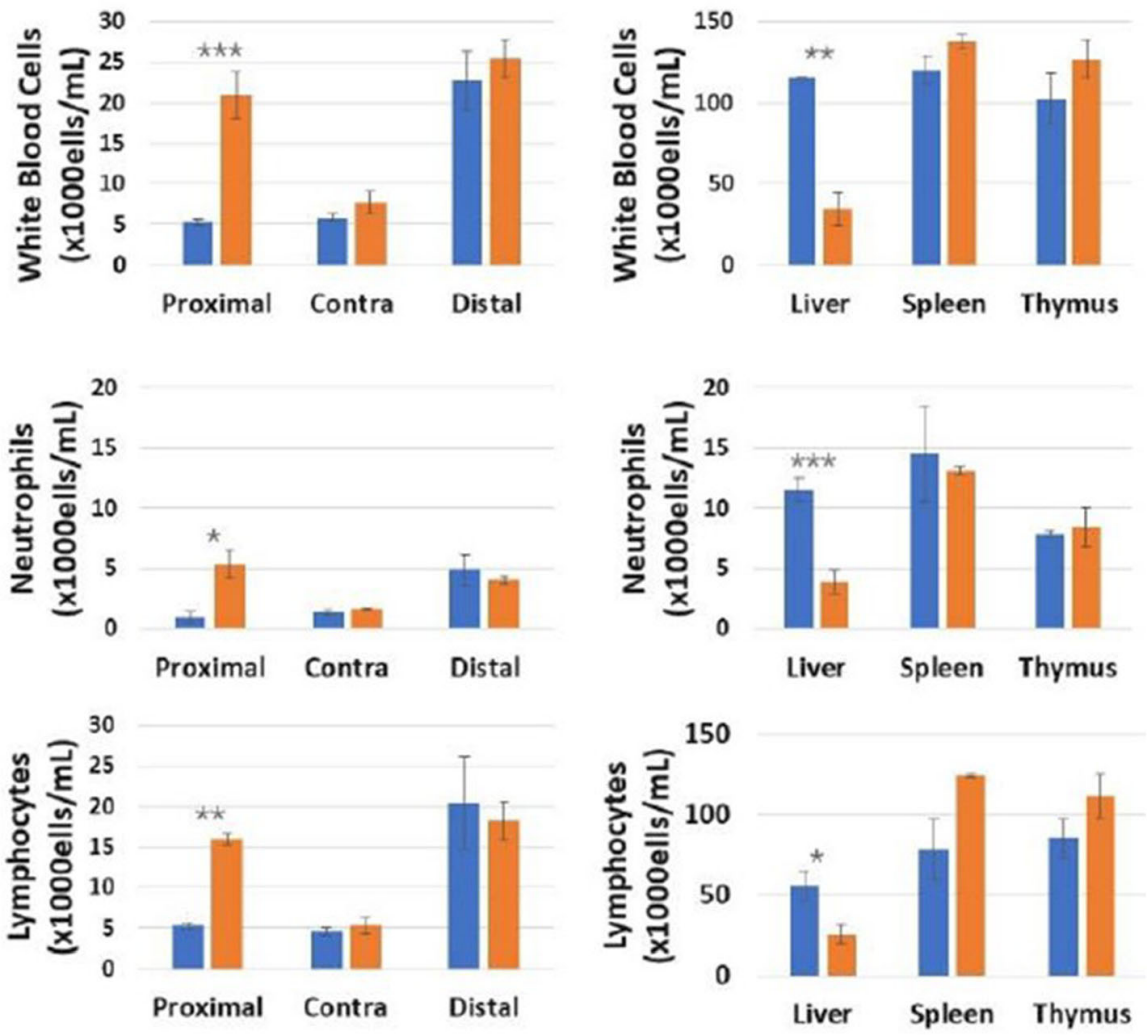
White blood cell, neutrophil and lymphocyte concentrations in the directly stimulated popliteal lymph node, contralateral popliteal lymph node, distal axillary lymph node, spleen liver, and lymphatic duct/lymphatic fluid with and without (sham CTRL) an ultrasound-induced nerve stimulation directly above the popliteal lymph node. *N* = 6. One way ANOVA with multiple comparison analysis * = *p* ≤ 0.05, ** = *p* ≤ 0.005,*** = *p* ≤ 0.0005; Ultrasound stimulation parameters: 1.1 MHz, 136.36 μs pulse length, and 0.5 ms pulse repetition period, applied pressure of 0.83 MPa as previously reported (Cotero et al., 2019b)

We tested whether focused, noninvasive stimulation of neural tissue directly within and around the popliteal LN also results in changes in WBC counts. After delivery of ultrasound neurostimulation (1.1 MHz acoustic frequency, 0.5 ms pulse repetition period, 0.27 duty cycle, 136.36 us pulse length, 0.83 MPa peak positive pressure) using a peripheral ultrasound neuromodulation device previously described (Cotero et al., 2019a; Cotero et al., 2019b; Puleo et al., 2019; Cotero et al., 2020) we found that there were similar effects on WBC counts as with direct electrical sciatic nerve stimulation in the targeted popliteal LN and the liver (Fig. 4). At the same time, the effects on WBC counts in the spleen and the contralateral LN were attenuated compared to those seen with direct nerve stimulation (Fig. 4).

### Effects of direct sciatic nerve stimulation on WBC counts in the presence of acute inflammation

**Fig. 5:**
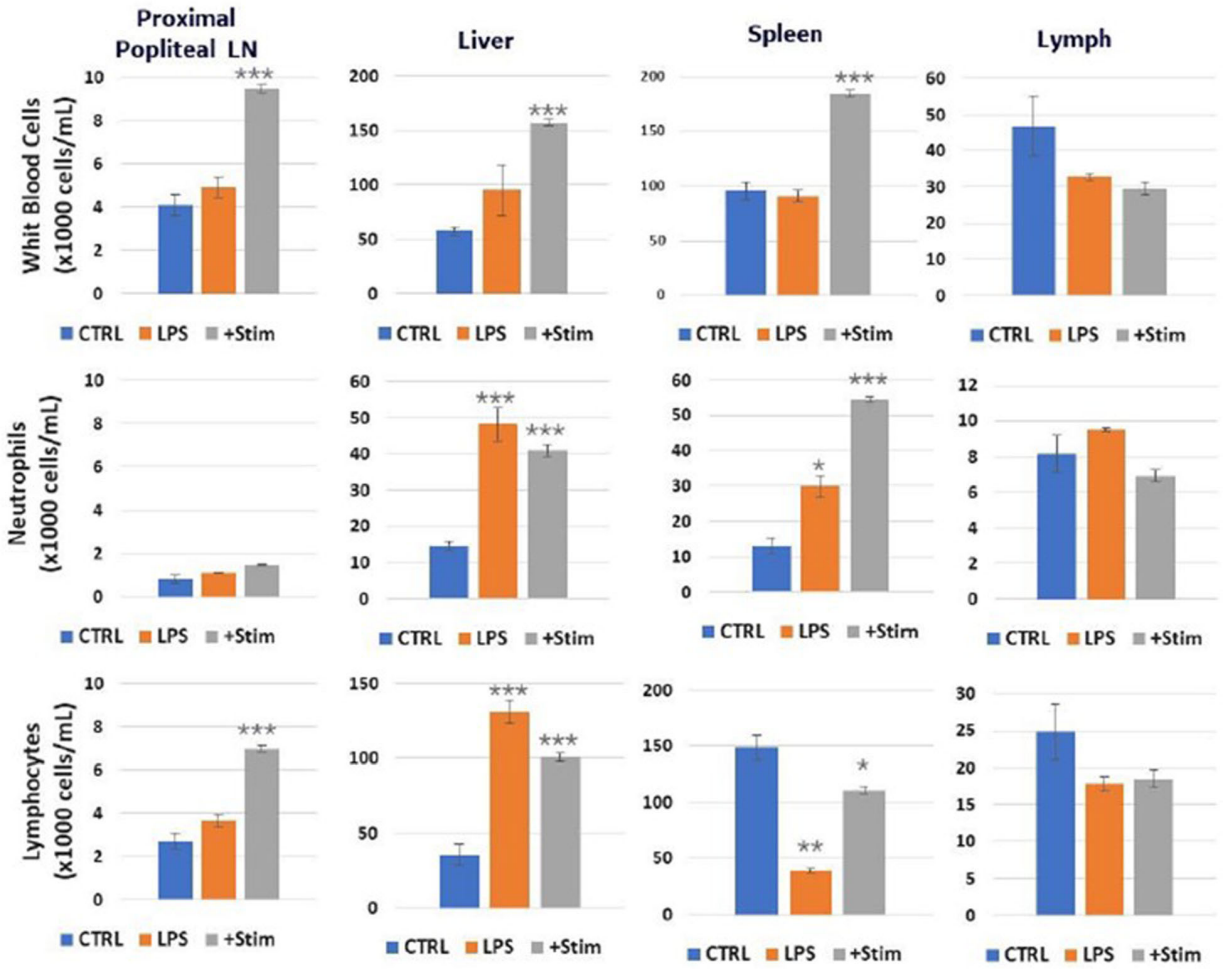
White blood cell, neutrophil and lymphocyte concentrations in the directly stimulated popliteal lymph node, liver, spleen, and lymphatic duct/fluid with (LPS + Stim) or without (LPS Only) electrical sciatic nerve stimulation after pre-exposure to lipopolysaccharide (LPS) compared to no LPS/sham stimulation (CTRL) controls. *N* = 5. One way ANOVA with multiple comparison analysis * = *p* ≤ 0.05, ** = *p* ≤ 0.005,*** = *p* ≤ 0.0005; Electrical stimulation parameters: 0.5 mA, 20 Hz, pulse length 50 msec

Finally, we documented the effects of direct sciatic nerve stimulation on WBC counts in the presence of acute inflammation, induced by LPS injection. LPS was given via intraperitoneal injection to animals at a dose of 10 mg/kg, which corresponds to an approximate LD75 dose and has previously been shown to result in systemic inflammation and metabolic dysfunction, peaking at 4 h post injection (Cotero et al., 2019b). Under these conditions, nerve stimulation did not result in reduction in WBC count in the liver (Fig. 5), in contrast to the decrease in WBC counts observed in the experiments without LPS (Fig. 2). Neutrophil counts were increased in both the liver and spleen upon injection of LPS, and this increase was not affected by the electrical nerve stimulation in the liver. In contrast, splenic neutrophil counts were further increased in the spleen upon electrical nerve stimulation at the lymph node site, demonstrating on additive effect of the LPS injection and nerve stimulation. This contrasts with the effects of nerve stimulation without prior LPS exposure (Fig. 2b), in which stimulation resulted in immobilization of neutrophils from the splenic store into lymph fluid and circulation. Lymphocyte counts were also increased in the liver after injection of LPS, and the increase was not affected by the electrical nerve stimulation. In contrast, lymphocyte counts were decreased in the spleen after LPS injection, and this effect was attenuated by the addition of the electrical stimulation. Unlike the effect at these distal sites, LPS injection had no effect on cell counts within the proximal lymph node, and electrical stimulation resulted in a similar increase in both neutrophils and lymphocytes with (Fig. 2a) or without (Fig. 5) the LPS injection. This data demonstrates that the effect of nerve stimulation on immune cell trafficking may be dependent on inflammatory state at the time of stimulation.

## Discussion

Mounting an adequate immune response requires interaction of multiple cell types from the tissue associated with the primary infection (or insult) and distal lymph tissue (Huston, 1997). For example, generation of an antibody response to a T-cell dependent soluble protein requires recognition of the antigen and interaction of the antigen-presenting cell (e.g. dendritic cells) with T-helper cells and B cells, which often takes place within secondary lymph organs. Lymph nodes and the spleen provide critical meeting points for immune cells and antigens, and it is therefore beneficial to promote increased interactions of targeted cells types responsible for mounting an immune defense. Networks of lymphatic vessels channel free and cell-borne antigen to lymph nodes, where they are further directed to LN compartments that are enriched for lymphocytes (Gonzalez et al., 2011). It has long been known that lymphatic flow rates are dynamic and are regulated by both cytokine/inflammatory and neurotransmitter mediators (Huxley & Scallan, 2011). However, more recently it has been demonstrated that lymphocyte egress rates from lymph nodes are also modulated by nerve signaling through the functional association between adrenergic and chemokine receptors (that are responsible for the gating kinetics at lymph node exits) (Nakai et al., 2014). This new finding provides a mechanism for nerve mediated changes in LN cellularity and promotion of increased interrogation of antigen by lymphocyte populations within the lymph compartment.

Herein we provide evidence that lymph nodes and other immune tissues may be interconnected by long range nerve pathways and/or reflexes, allowing communication for coordinated activity. We show that upon stimulation of the nerves entering a local lymph node (i.e. both efferent and afferent neurons from that site) there are concurrent changes in neurotransmitter signaling and immune cell trafficking in both the local lymph node and distal immune sites. Interestingly, this signaling to distal immune tissue is site dependent on tissue location (i.e. there is no signaling to distal lymph nodes far away from the stimulation site) and appears targeted to specific organs (e.g. liver and spleen). In addition, the changes in immune cell trafficking at different sites (i.e. liver versus spleen) were coincident with different neurotransmitter signals, suggesting that the neuroimmune system is hard-wired to interact with different immune cell sub-types at different locations or based on different sensory inputs.

Norepinephrine and epinephrine function as both neurotransmitters and neurohormones (Marino & Cosentino, 2013; Feher, 2012). As a neurotransmitter, release from nerve endings enables communication to local immune cells via axoextracellular synaptic transmission. As a neurohormone, secretion into the blood stream by chromaffin cells in the adrenal medulla enables communication to systemic immune cells via the circulation. In the periphery, the primary source of norepinephrine is secretion by peripheral nerves; however, epinephrine is primarily secreted by the adrenal gland (Marino & Cosentino, 2013; Feher, 2012). Our results show differential concentrations of the two neurotransmitter across the different immune compartments after stimulation (Fig. 1c). It is possible that the kinetics of epinephrine release, re-uptake, and degradation are such that transient secretion from the adrenal gland results in different concentrations of the neurohormone across tissues/compartments following a stimulus. However, there are also other non-adrenal sources of epinephrine that may be involved, these include sympathetic co-transmission from peripheral nerves (which is thought to occur only after sustained activation and continuous release of norepinephrine (Schlaich & Esler, 2004)) and stimulated release from immune cell stores of epinephrine within the local site (Marino & Cosentino, 2013). Significant evidence for the existence of a classical pathway for catecholamine synthesis exists in rodent and human immune cells, including the expression of tyrosine hyodroxylase, increased intracellular concentrations of catecholamine in vitro upon mitogenic-stimulation, and prevention of intracellular catecholamine changes following application of thyrosine hydroxylase inhibitors (Marino & Cosentino, 2013). Due to these multiple potential mechanisms for differential neurotransmitter/neurohormone response to the stimulus and subsequent modulation of tissue cellularity, further experiments are warranted to determine the extent of local versus systemic mechanisms controlling immune cell distribution in response to stimuli. It is also important to note that immune cell counting measurements were obtained in this manuscript using electrical stimulation parameters chosen due to the optimal response in both norepinephrine and epinephrine in the proximal popliteal lymph node (Fig. S1 and S2). It is possible that stimulation using the alternative amplitudes and frequencies shown in the supplemental figures may affect/stimulate the sources of the neurotransmitters/neurohormones (e.g. norepinephrine and epinephrine) uniquely leading to an amplitude or frequency specific immune response. Additional examination of the effect of different stimulation parameters on whole body immune cellularity should be a focus of future work.

The potential implications for orchestrated nerve-mediated control of immune cellularity across the body are significant, as organisms typically contain only 10–100 s of naïve antigen specific lymphocytes per epitope (Altman et al., 1996). Therefore, mounting an immune response to that epitope requires an antigen presenting cell (APC) to screen through 100,000 s of cells to locate an epitope match. Interestingly, it is well documented that (in addition to differential expression across immune cell types) there is a higher level of expression of adrenergic receptors on naïve versus stimulated/activated lymphocytes, perhaps providing a mechanism to promote interaction of naïve cells with APCs in activated lymph nodes (i.e. lymph nodes receiving a high level of nerve signaling/activation) (Gonzalez et al., 2011; Hanes et al., 2016). Infection, tissue injury, and inflammation are all known to increase firing rates within surrounding sympathetic nerves (Pongratz & Straub, 2014), which in turn increase release of catecholamines in surrounding lymphoid organs and tissues. These firing rates may be confined to specific anatomical locations (as in the case of localized infections or insults) or be associated with a system wide alteration in sympathetic nerve activity (as in the case of high stress situations or broad systemic infections). The data shown herein suggest that nerve connections between lymph organs and compartments may provide the immune system with a method of rapidly altering immune cell distribution across the organism, as it responds to insults at different locations or of different intensities.

The nerve resection experiments (Fig. S3) were performed above the stimulation electrodes; therefore, the nerve path between the stimulation site and the targeted proximal popliteal lymph node remained intact. This may allow for local efferent, but not afferent signaling within the resected nerve. In addition, resection was confined to the sciatic nerve, leaving other possible afferent pathways intact (such as the descending cutaneous nerve), which may account for the differences between the lidocaine (Figs. 1 and 2) and nerve resection experiments (Fig. S3). These differences suggest that the nerves governing neutrophil versus lymphocyte trafficking within the liver originate from different sensory fields and/or neural pathways. The ultrasound stimulation experiments provide further support of the existence of multiple pathways controlling immune cell trafficking across the different compartments. The more targeted ultrasound stimulus (i.e. ultrasound stimulation only on neurons within the focused ultrasound field versus the entire nerve branch using the electrical techniques) resulted in a different pattern of immune cell trafficking. Specifically, the changes in cellularity within the lymph node and liver were similar to the electrical stimulator experiments; however, the changes in splenic trafficking and contralateral LN were attenuated. Ultrasound-mediated peripheral nerve modulation is a new tool that has recently been shown by our group to activate known peripheral nerve pathways (i.e. splenic cholinergic anti-inflammatory reflex) with similar outcomes (i.e. neurotransmitter secretion and effect on resident splenic immune cells) to traditional implant-based stimulation (Cotero et al., 2019a; Cotero et al., 2019b; Puleo & Cotero, 2020; Cotero et al., 2020). Our team has also recently shown the ultrasound stimulus to result in neuromodulation across several other (i.e. non-splenic) sites of peripheral nerve innervation, and characterized the ultrasound effect on peripheral nerve activity in both in vivo and in vitro platforms (Cotero et al., 2020). The ultrasound stimulation parameters used herein were based on the stimulation parameters shown to achieve optimal neurotransmitter release and nerve activation in the previous studies (Cotero et al., 2019b; Cotero et al., 2020); however, future studies on the specific effects and differences in effects of ultrasound and implant-based modulation of the lymphatic system (including the types of levels of neurotransmitters modulated in each compartment) are warranted in future studies.

Under normal physiological conditions sympathetic-immune cell signaling is thought be involved with activation of the inflammatory response to foreign antigens (Gunasekaran et al., 2018; Pongratz & Straub, 2014; Elenkov et al., 2000). However, the same nerves are also implicated in the subsequent restoration of homeostasis and recovery (Scanzano & Cosentino, 2015; Lorton & Bellinger, 2015). Presumably these different roles are performed using the same neurotransmitter and neuropeptide repertoire. Therefore, neuro-immune interactions must be flexible and provide both up and down-regulation of various immune cell functions (including expansion, differentiation, and cytokine secretion). The mechanisms that provide this flexibility are the diverse set of intercellular pathways that mediate immune cell function upon neurotransmitter binding to their resident receptors. During adrenergic signaling, immune cells can operate through both canonical (e.g. neurotransmitter binding results in induction cAMP and PKA pathways) or non-canonical pathways depending on the status of their current environment (Lorton & Bellinger, 2015). Within this non-canonical pathway set, several G protein kinases are known to result in desensitization of adrenergic receptors through phosphorylation of specific serines on the neurotransmitter receptor and prevention of receptor signaling through cAMP. In addition, the conformational changes associated with this phosphorylation results in binding of the protein B-arrestin and internalization of the receptors back into cytosol (Krasel et al., 2005). It is important to point out that the results on nerve-mediated changes in immune cellularity may be dependent on use of the short-term (i.e. 3 min long) stimulus duration used herein, and that stimulation over a longer period of time may result in different neuroimmune effects, as a result of the compensatory mechanisms discussed above or other specific effects of long-term versus short-term neurotransmitter signaling (Lorton & Bellinger, 2015; Wong et al., 2012).

In agreement with previous studies that show altered immune cell response to neurotransmitter signaling based on changing immune system conditions, we observed an environment- or state-dependent alteration in immune cell distribution upon nerve stimulation. That is, some changes in immune cell distribution that occurred in the non-LPS animals (Figs. 1 and 2) were abolished when experiments were performed in the presence of systemic inflammation (Fig. 5; caused by injection of the endotoxin, LPS). LPS is known to cause wide-spread modulation of the sympathetic nervous system (Elenkov et al., 2000; Ağaç et al., 2018; Kox et al., 2014), and therefore pre-stimulation exposure of the animal to LPS may trigger a system-wide change in inflammation and response. Increased nerve activity prior to stimulation may cause desensitization of neuro-immune signaling (through the pathways discussed above (Lorton & Bellinger, 2015; Krasel et al., 2005) or others (Ağaç et al., 2018)). In an immune system context, the immune cell mobilization (i.e. release of immune cells from the spleen and liver) that occurred during lymphatic nerve stimulation in the non-LPS treated animals may be beneficial in enabling an increase in lymphocyte-APC interactions during a local insult. However, mobilization of immune cells during a systemic infection or exposure may move essential immune cells away from those vital organs, requiring a different response depending on the insult intensity. It is interesting to note that the different sources of neurotransmitters described above (i.e. adrenal, neuronal, and immune cell sources) may be affected differently by various stimuli (i.e. local versus systemic autonomic nerves activation, short-term versus long-term activation of neurons, or purely neural versus neural/inflammatory stimuli), and result in physiological state dependent neuroimmune signaling repertoires. Recently, the first evidence of a “state-based” neuroimmune response has been reported to modulate B cell/plasma cells differentially based on that activity sate of the glucocorticoid axis (Zhang et al., 2020), and it is likely that similar state-dependent control mechanisms exist across the neuroimmune system.

## Conclusions

Herein, we demonstrate that targeted stimulation of the nerves projecting to local lymph nodes results in transient increase of neurotransmitter/neuropeptide concentrations, and subsequent changes in immune cell cellularity at that site. This is in agreement with recent reports demonstrating that lymphocyte egress from lymphatic tissue is modulated by the nervous system through associations between adrenergic and chemokine receptors, and that the rate of immune cell gating kinetics is influenced by local sympathetic nerve activity (Luster et al., 2005). Surprisingly, we demonstrate herein that long-range nerve pathways may exist enabling coordinated changes in immune system cellularity between these local lymph nodes sites, and distal lymph and immune organs. Additional studies will be necessary to elucidate the exact mapping and utility of these long-range nerve connection between lymph organs. However, the use of non-invasive tools to stimulate these pathways (such as minimally invasive sub-cutaneous electrodes or the non-invasive ultrasound stimulator used above) will enable further study and potential clinical application in vaccination, allergy, and auto-immune disease applications.

## Supplementary information

**Additional file 1: Figure S1.** Additional measures of neurotransmitter (norepinephrine, epinephrine, dopamine) and neuropeptide (neuropeptide Y (NPY), Substance P, and vasoactive intestinal peptide (VIP)) concentrations at the directly stimulated popliteal lymph node after stimulation with different stimulation frequencies (0, 0.5, 20, 200, and 3000 Hz; at 0.5 mA intensity and pulse length of 50 msec). *N* = 5, One way ANOVA with multiple comparison analysis * = *p* ≤ 0.05, ** = *p* ≤ 0.005,*** = *p* ≤ 0.0005. **Figure S2.** Additional measures of neurotransmitter (norepinephrine, epinephrine, dopamine) and neuropeptide (neuropeptide Y (NPY), Substance P, and vasoactive intestinal peptide (VIP)) concentrations at the directly stimulated popliteal lymph node after stimulation with different intensities (0, 0.5, 2, 5, 7, and 10 mA; at 20 Hz frequency and pulse length of 50 msec). *N* = 5. One way ANOVA with multiple comparison analysis * = *p* ≤ 0.05, ** = *p* ≤ 0.005,*** = *p* ≤ 0.0005. **Figure S3.** Additional data comparing the concentration of neutrophils and lymphocytes within the directly stimulated lymph node, contralateral lymph node, distal axillary lymph node and distal immune tissue (liver, spleen, lymphatic duct/fluid) with stimulation following sciatic nerve resection (SN resection) directly above the site of electrical stimulation (along with previous data showing cell concentrations with (stimulated) or without (CTRL) stimulation and with stimulation following lidocaine injection). Electrical stimulation parameters: 0.5 mA, 20 Hz, pulse length 50 msec. **Figure S4.** An image of the bipolar electrodes created from electroacupuncture needles that were insulated using biocompatible epoxy (see materials and methods for details) up to the metal/stimulating tips. **Figure S5.** A schematic diagram of the custom voltage to current circuit built (within the current source stimulator system). The circuit was driven by an analog output from a data acquisition and analysis system (MP150 Biopac Systems), and the custum circuit provides a current output of approximately 1 mA per 1 V input (including output current and voltage monitoring). **Figure S6.** An example biphasic pulse from the system (S5) that was used during stimulation experiments; the pulse was constructed with a positive output (0.2 ms), no output (0.2 ms), then a negative output (0.2 ms), followed by a period of no output which was adjustable to change the effectively stimulation frequency. For example, a 49.4 ms pause would provide a total stimulation frequency of 20 Hz. **Figure S7.** Example output from impedance tests of the electrodes built for the experiment (shown above; details in materials and methods). The average electrode impedance ranged from 1200 to 1600 Ω.

## Data Availability

Supplemental figures and captions S1-S7 are included with this manuscript and available online.

## Abbreviations

CNS: Central nervous system
CCR7: C-C chemokine receptor type 7
CXC4: C-X-C chemokine receptor type 4
LN: Lymph node
mA: Milliamperes
Hz: Hertz
LC: Lidocaine
WBC: White Blood Cells
APC: Antigen presenting cell
cAMP: Cyclic adenosine monophosphate
PKA: Protein kinase A
LPS: Lipopolysaccharide
