# Supplementary material for: Evidence of Long-range nerve pathways connecting and coordinating activity in secondary lymph organs
Source: Bioelectron Med. 2020 Oct 23;6:21. doi: 10.1186/s42234-020-00056-2 (PMC7584093; doi:10.1186/s42234-020-00056-2)
Supplement: Supplementary file 1 — Additional file 1: Figure S1. Additional measures of neurotransmitter (norepinephrine, epinephrine, dopamine) and neuropeptide (neuropeptide Y (NPY), Substance P, and vasoactive intestinal peptide (VIP)) concentrations at the directly stimulated popliteal lymph node after stimulation with different stimulation frequencies (0, 0.5, 20, 200, and 3000 Hz; at 0.5 mA intensity and pulse length of 50 msec). N = 5, One way ANOVA with multiple comparison analysis * = p ≤ 0.05, ** = p ≤ 0.005,*** = p ≤ 0.0005. Figure S2. Additional measures of neurotransmitter (norepinephrine, epinephrine, dopamine) and neuropeptide (neuropeptide Y (NPY), Substance P, and vasoactive intestinal peptide (VIP)) concentrations at the directly stimulated popliteal lymph node after stimulation with different intensities (0, 0.5, 2, 5, 7, and 10 mA; at 20 Hz frequency and pulse length of 50 msec). N = 5. One way ANOVA with multiple comparison analysis * = p ≤ 0.05, ** = p ≤ 0.005,*** = p ≤ 0.0005. Figure S3. Additional data comparing the concentration of neutrophils and lymphocytes within the directly stimulated lymph node, contralateral lymph node, distal axillary lymph node and distal immune tissue (liver, spleen, lymphatic duct/fluid) with stimulation following sciatic nerve resection (SN resection) directly above the site of electrical stimulation (along with previous data showing cell concentrations with (stimulated) or without (CTRL) stimulation and with stimulation following lidocaine injection). Electrical stimulation parameters: 0.5 mA, 20 Hz, pulse length 50 msec. Figure S4. An image of the bipolar electrodes created from electroacupuncture needles that were insulated using biocompatible epoxy (see materials and methods for details) up to the metal/stimulating tips. Figure S5. A schematic diagram of the custom voltage to current circuit built (within the current source stimulator system). The circuit was driven by an analog output from a data acquisition and analysis system (MP150 Biopac [file 42234_2020_56_MOESM1_ESM.docx]

S1


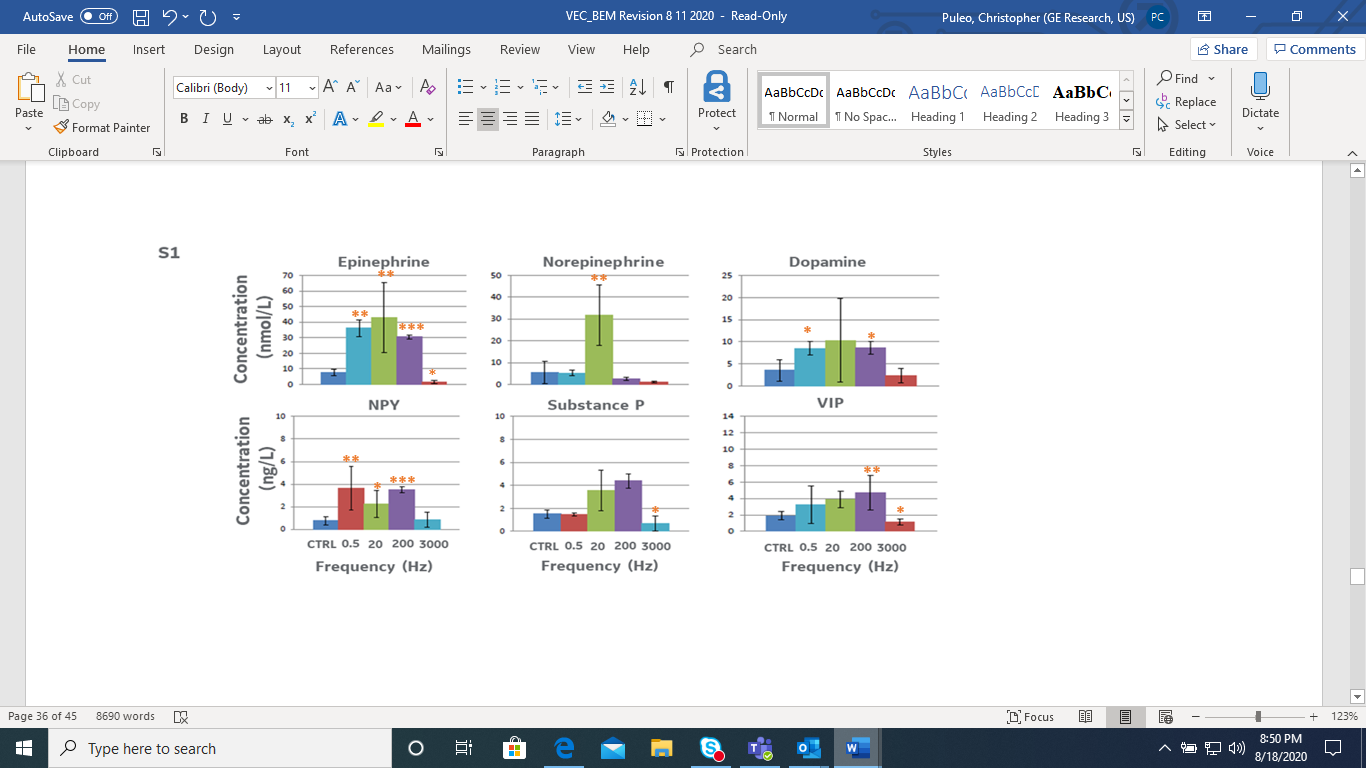


S1. Additional measures of neurotransmitter (norepinephrine, epinephrine, dopamine) and neuropeptide (neuropeptide Y (NPY), Substance P, and vasoactive intestinal peptide (VIP)) concentrations at the directly stimulated popliteal lymph node after stimulation with different stimulation frequencies (0, 0.5, 20, 200, and 3000 Hz; at 0.5 mA intensity and pulse length of 50 msec). N=5, One way ANOVA with multiple comparison analysis *=p≤0.05, **=p≤0.005,***=p≤0.0005.

S2


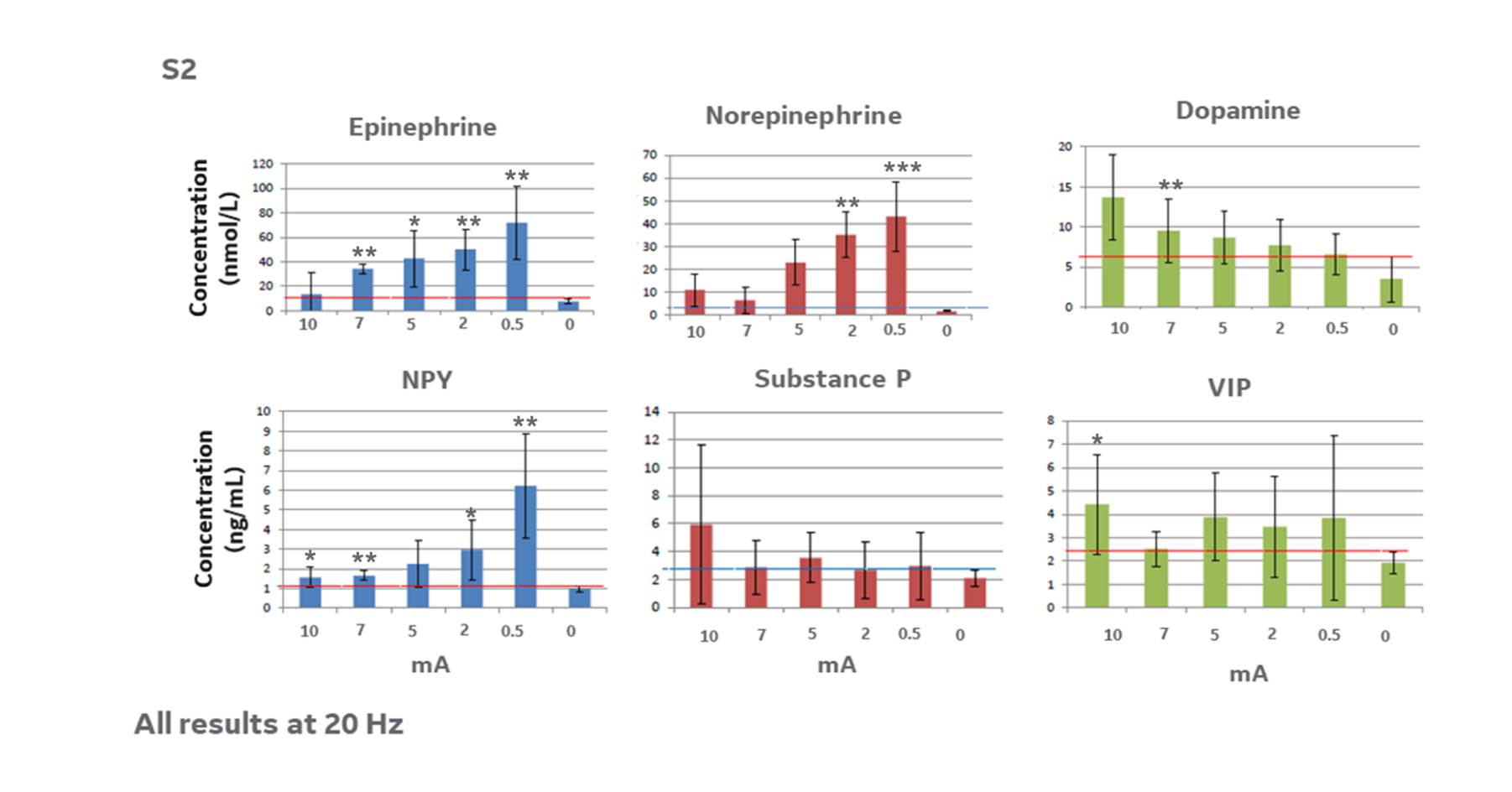


S2. Additional measures of neurotransmitter (norepinephrine, epinephrine, dopamine) and neuropeptide (neuropeptide Y (NPY), Substance P, and vasoactive intestinal peptide (VIP)) concentrations at the directly stimulated popliteal lymph node after stimulation with different intensities (0, 0.5, 2, 5, 7, and 10 mA; at 20 Hz frequency and pulse length of 50 msec). N=5. One way ANOVA with multiple comparison analysis *=p≤0.05, **=p≤0.005,***=p≤0.0005.

S3


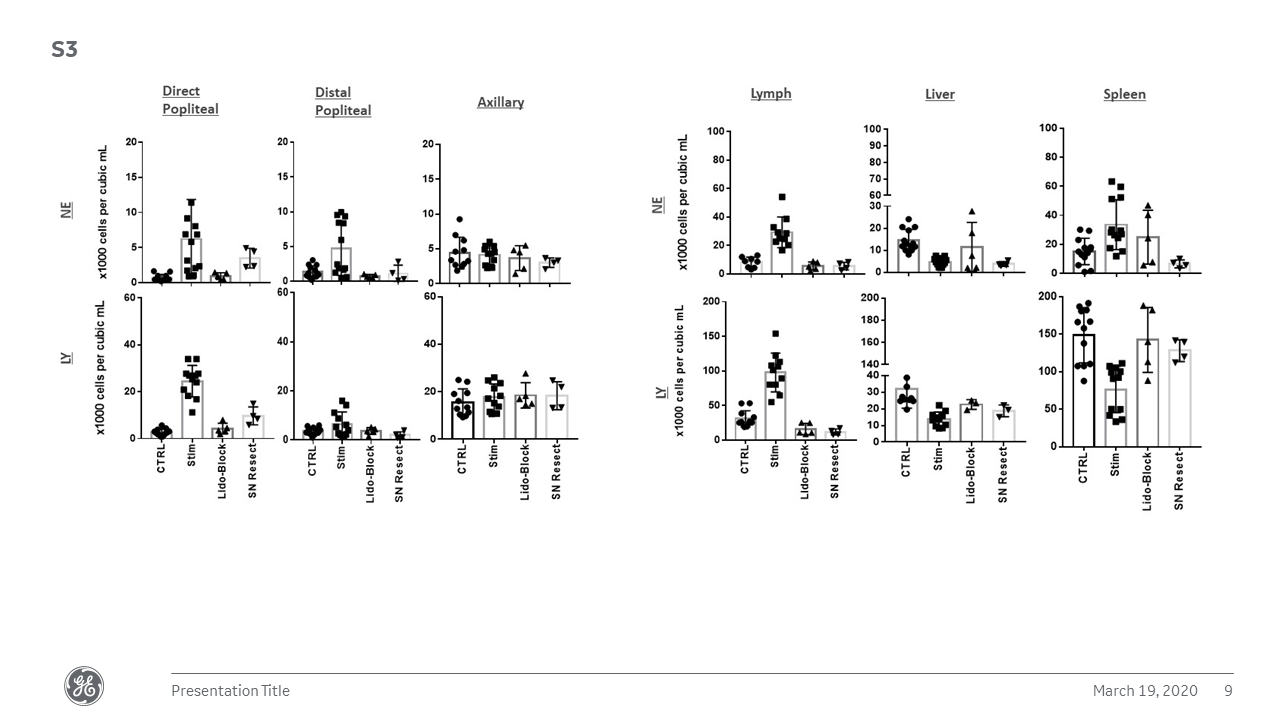


S3. Additional data comparing the concentration of neutrophils and lymphocytes within the directly stimulated lymph node, contralateral lymph node, distal axillary lymph node and distal immune tissue (liver, spleen, lymphatic duct/fluid) with stimulation following sciatic nerve resection (SN resection) directly above the site of electrical stimulation (along with previous data showing cell concentrations with (stimulated) or without (CTRL) stimulation and with stimulation following lidocaine injection). Electrical stimulation parameters: 0.5 mA, 20 Hz, pulse length 50 msec.

S4


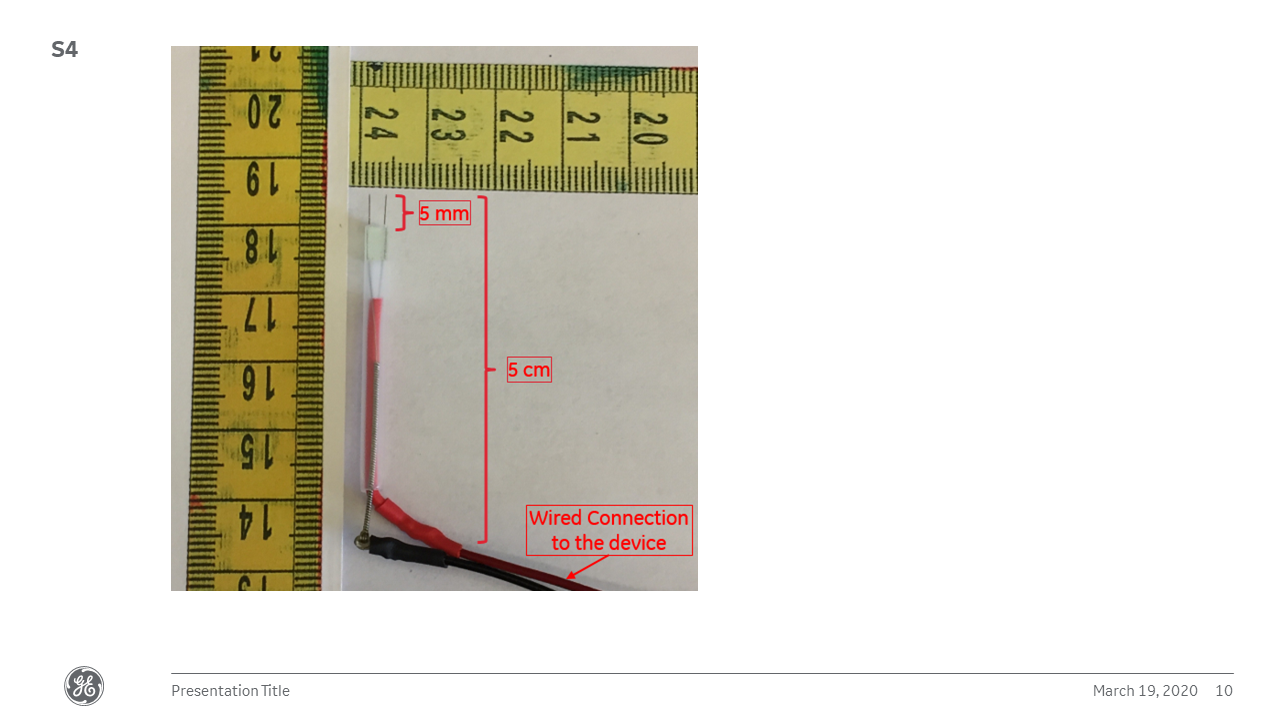


S4. An image of the bipolar electrodes created from electroacupuncture needles that were insulated using biocompatible epoxy (see materials and methods for details) up to the metal/stimulating tips.

S5


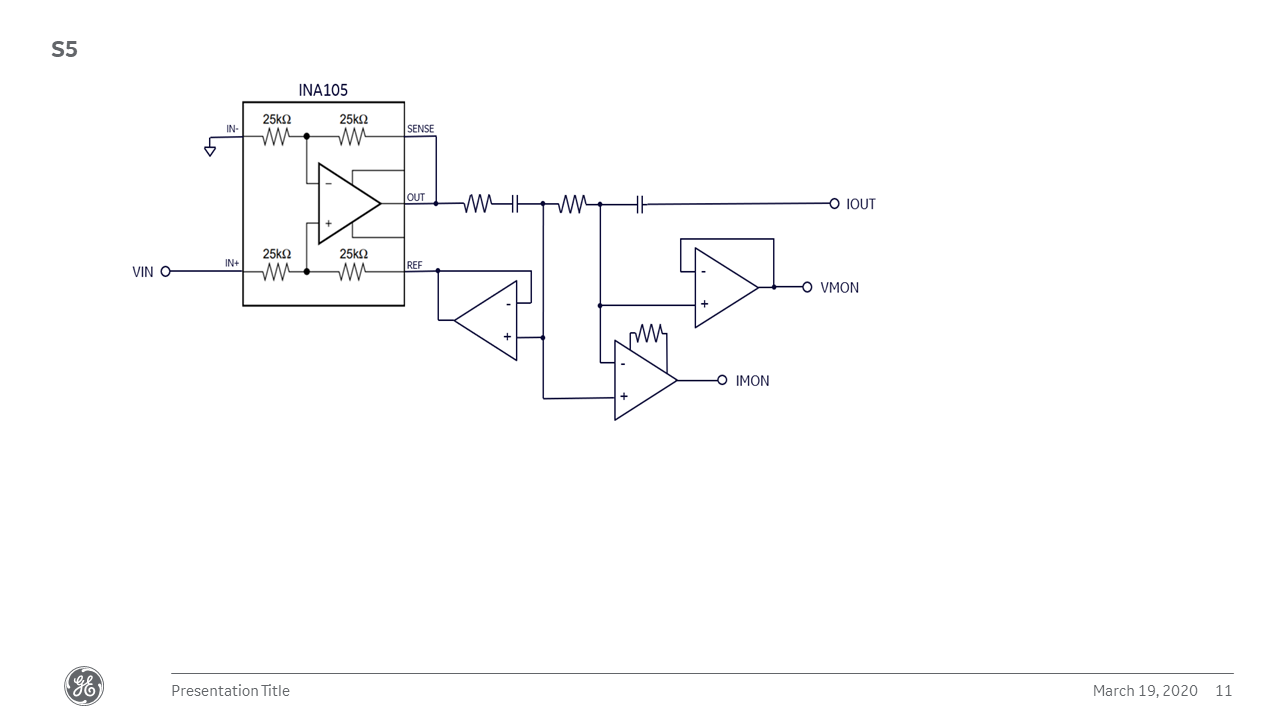


S5. A schematic diagram of the custom voltage to current circuit built (within the current source stimulator system). The circuit was driven by an analog output from a data acquisition and analysis system (MP150 Biopac Systems), and the custum circuit provides a current output of approximately 1 mA per 1V input (including output current and voltage monitoring).

S6


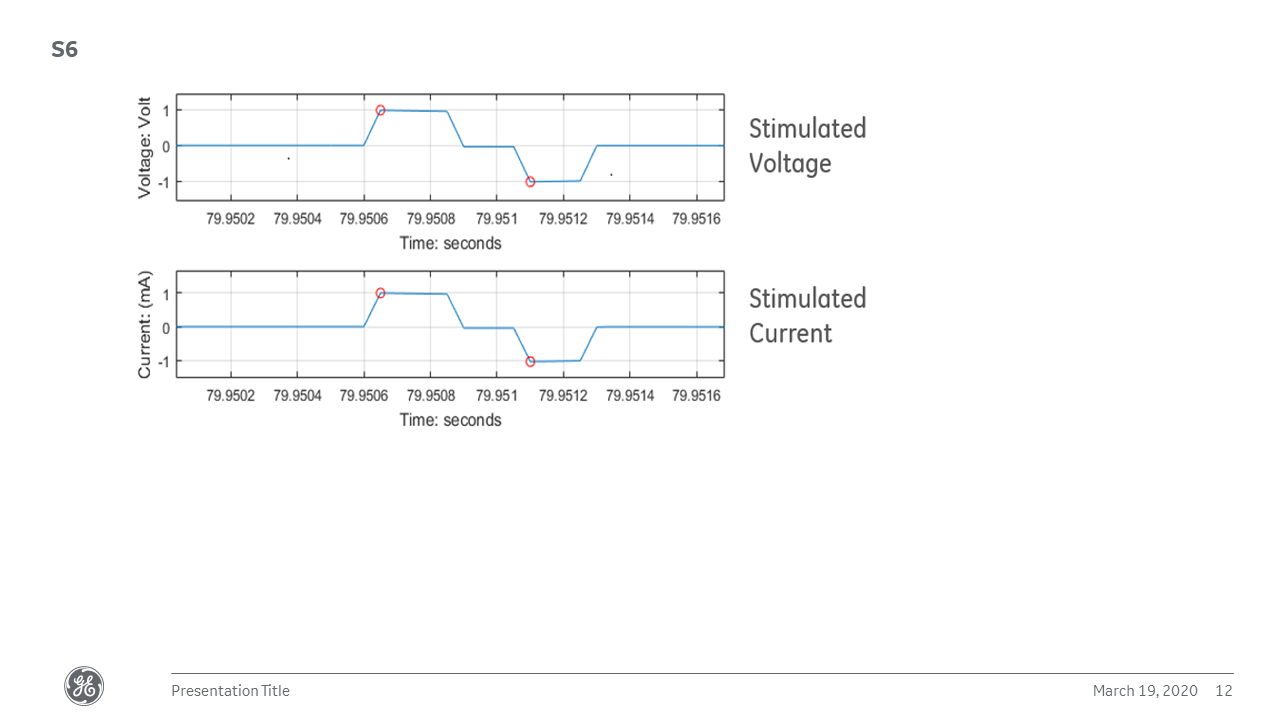


S6. An example biphasic pulse from the system (S5) that was used during stimulation experiments; the pulse was constructed with a positive output (0.2 ms), no output (0.2 ms), then a negative output (0.2 ms), followed by a period of no output which was adjustable to change the effectively stimulation frequency. For example, a 49.4 ms pause would provide a total stimulation frequency of 20 Hz.

S7


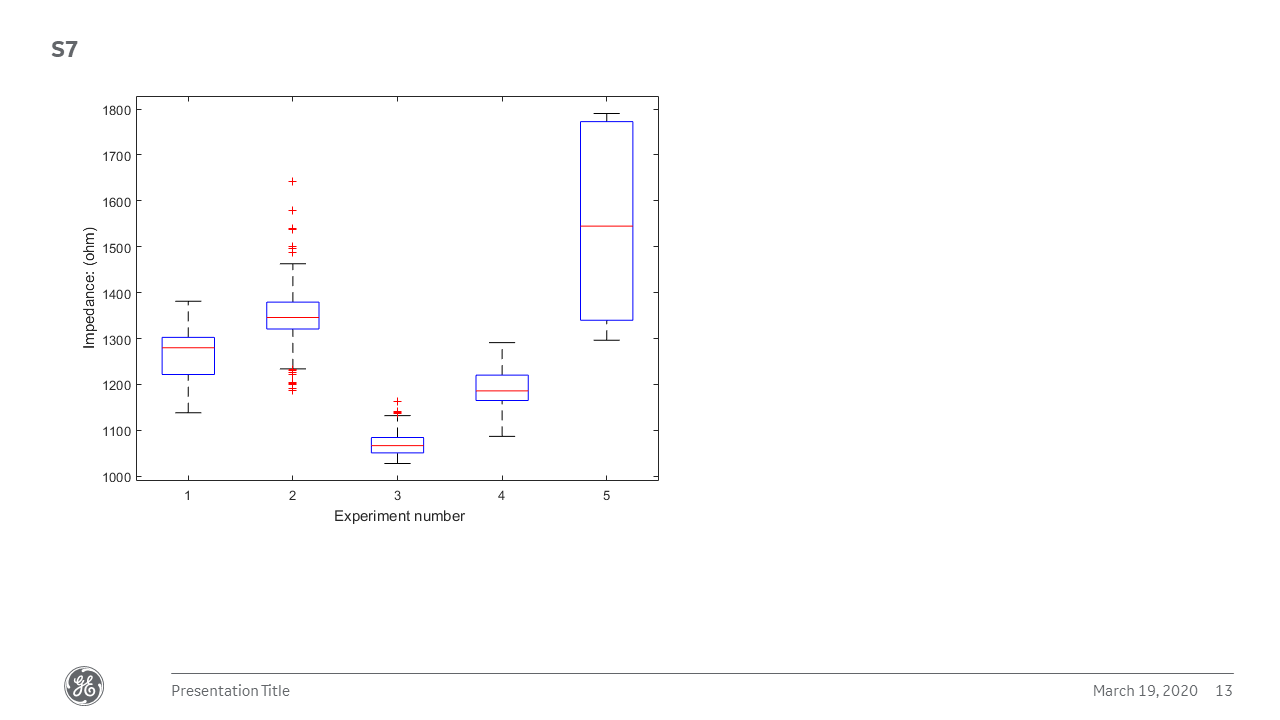


S7. Example output from impedance tests of the electrodes built for the experiment (shown above; details in materials and methods). The average electrode impedance ranged from 1200 to 1600 ohms.
